# Supplementary material for: Information and Communications Technology (ICT) Usage during COVID-19: Motivating Factors and Implications
Source: Int J Environ Res Public Health. 2021 Mar 30;18(7):3571. doi: 10.3390/ijerph18073571 (PMC8036312; doi:10.3390/ijerph18073571)
Supplement: Supplementary file 1 [file ijerph-18-03571-s001.pdf]

Appendix A

[The Need to Belong Scale]

1. Please indicate the degree to which each statement is true or characteristic of you on a 5-point scale: 1 = not at all, 2 = slightly, 3 = moderately, 4 = very, 5 = extremely

1. If other people don't seem to accept me, I don't let it bother me.
2. I try hard not to do things that will make other people avoid or reject me.
3. I seldom worry about whether other people care about me.
4. I need to feel that there are people I can turn to in times of need.
5. I want other people to accept me.
6. I do not like being alone.
7. Being apart from my friends for long periods of time does not bother me.
8. I have a strong "need to belong."
9. It bothers me a great deal when I am not included in other people's plans.
10. My feelings are easily hurt when I feel that others do not accept me.

[Fear of Missing Out Scale]

2. Below is a collection of statements about your everyday experience. Please indicate how true each statement is of your general experiences on a 5-point scale: 1 = Not at all true of me, 2 = Slightly true of me, 3 = Moderately true of me, 4 = Very true of me, 5 = Extremely true of me. Please answer according to what really reflects your experiences rather than what you think your experiences should be. Please treat each item separately from every other item.

1. I fear others have more rewarding experiences than me.
2. I fear my friends have more rewarding experiences than me.
3. I get worried when I find out my friends are having fun without me.
4. I get anxious when I don't know what my friends are up to.
5. It is important that I understand my friends ''in jokes''.
6. Sometimes, I wonder if I spend too much time keeping up with what is going on.
7. It bothers me when I miss an opportunity to meet up with friends.
8. When I have a good time it is important for me to share the details online (e.g. updating status).
9. When I miss out on a planned get-together it bothers me.
10. When I go on vacation, I continue to keep tabs on what my friends are doing.

[Perceived Attachment to Phones Scale]

3. Please indicate the degree to which you agree with each statement on a 5-point scale: 1 = strongly disagree, 2 = somewhat disagree, 3 = neutral/undecided, 4 = somewhat agree, 5 = strongly agree

1. I would feel uncomfortable if I didn't have my phone with me for a long period of time
2. I would feel lost if I didn't have a cell phone
3. I would feel detached from my friends if I didn't have a cell phone
4. I feel momentarily distressed if I realize that I am without my phone while I am out and about
5. I would rather lose my wallet than my phone

[Habitual Smartphone/Internet Behavior Scale]

4. Please indicate the degree to which you agree with each statement on a 5-point scale: 1 = strongly disagree, 2 = somewhat disagree, 3 = neutral/undecided, 4 = somewhat agree, 5 = strongly agree

1. Smartphone usage is part of my daily routines
2. Internet usage is part of my daily routines
3. Using my smartphone has become a habit to me
4. Using the Internet has become a habit to me
5. I use my smartphone automatically
6. I use the Internet automatically
7. It's a habit of mine to use my smartphone
8. It's a habit of mine to use the Internet
9. My smartphone is a part of my life
10. The Internet is a part of my life
11. When I need to complete a certain task, the use of my smartphone is an obvious choice
12. When I need to complete a certain task, the use of the Internet is an obvious choice
13. I normally use my smartphone without explicitly planning to do so
14. I normally use the Internet without explicitly planning to do so

[Self Regulation Scale]

5. Please indicate the degree to which each statement is true or characteristic of you on a 4-point scale: 1 = not at all true, 2 = barely true, 3 = somewhat true, 4 = completely true

1. I can concentrate on one activity for a long time, if necessary
2. If I am distracted from an activity, I don't have any problem coming back to the topic quickly
3. If an activity arouses my feelings too much, I can calm myself down so that I can continue with the activity soon
4. If an activity requires a problem-oriented attitude, I can control my feelings
5. It is difficult for me to suppress thoughts that interfere with what I need to do
6. I can control my thoughts from distracting me from the task at hand
7. When I worry about something, I cannot concentrate on an activity
8. After an interruption, I don't have any problem resuming my concentrated style of working
9. I usually have a whole bunch of thoughts and feelings that interfere with my ability to work in a focused way
10. I stay focused on my goal and don't allow anything to distract me from my plan of action

[Boredom Proneness Scale]

6. Please indicate the degree to which you agree with each statement on a 7-point scale: 1 (strongly disagree) to 7 (strongly agree)

1. It is easy for me to concentrate on my activities
2. I find it easy to entertain myself
3. I get a kick out of most things I do
4. In any situation I can usually find something to do or see to keep me interested

5. Many people would say that I am a creative or imaginative person
6. Among my friends, I am the one who keeps doing something the longest
7. Having to look at someone's home movies or travel slides bores me tremendously
8. Many things I have to do are repetitive and monotonous
9. It would be very hard for me to find a job that is exciting enough
10. Unless I am doing something exciting, even dangerous, I feel half-dead and dull
11. It seems that the same old things are on television or the movies all the time; it's getting old
12. When I was young, I was often in monotonous and tiresome situations

[Abbreviated version of the Big Five Inventory]

7. Please indicate the degree to which you agree with each statement on a 5-point scale: 1 = disagree strongly, 2 = disagree a little, 3 = neither agree nor disagree, 4 = agree a little, 5 = agree strongly

How well do the following statements describe your personality? I see myself as someone who …

1. is reserved
2. is generally trusting
3. tends to be lazy
4. is relaxed, handles stress well
5. has few artistic interests
6. is outgoing, sociable
7. tends to find fault with others
8. does a thorough job
9. gets nervous easily
10. has an active imagination
11. is considerate and kind to almost everyone

[ICT Questionnaire]

The following questions are about Information and Communications Technology (ICT). This kind of technology is defined as the integration of telecommunications and computers as well as necessary software, hardware, and audiovisual systems that enable users to access, store, transmit, and manipulate information and to communicate in a digital form.

8. Before the pandemic: What were the major ICT devices did you use on a daily basis at home? (check all that apply)
    - Computer for non-Internet use
    - Computer for Internet use (e.g., email, search engine, social media)
    - Cable TV
    - Smart TV (e.g., Netflix)
    - Tablet
    - Smartwatch
    - Video games (stand along or computer-based)
    - Landline phone
    - Smartphone
    - Mobile phone (not smartphone)

- Data storage system
- Other, please specify ______

9. Now during the pandemic: What were the major ICT devices did you use on a daily basis at home? (check all that apply)
    - Computer for non-Internet use
    - Computer for Internet use (e.g., email, search engine, social media)
    - Cable TV
    - Smart TV (e.g., Netflix)
    - Tablet
    - Smartwatch
    - Video games (stand along or computer-based)
    - Landline phone
    - Smartphone
    - Mobile phone (not smartphone)
    - Data storage system
    - Other, please specify ______

10. Before the pandemic: On average, how many hours did you spend on Information and Communications Technology for getting news and knowing what's happening on a daily basis? _____ hours

11. Now during the pandemic: On average, how many hours did you spend on Information and Communications Technology for getting news and knowing what's happening on a daily basis? _____ hours

12. Before the pandemic: from which sources did you get news and updates about what's happening in general on a daily basis? (check all that apply)
    - TV news channels
    - Newspapers and magazines
    - Radio
    - Emails from federal/state/local organizations
    - Emails from school districts
    - Searchable websites
    - Social media
    - Friends and family members
    - Other, please specify ______
    - Other, please specify ______

13. Now during the pandemic: from which sources did you get news and updates about what's happening in general on a daily basis? (check all that apply)
    - TV news channels
    - Newspapers and magazines
    - Radio
    - Emails from federal/state/local organizations
    - Emails from school districts
    - Searchable websites
    - Social media

- Friends and family members
- Other, please specify ______
- Other, please specify ______

[Reactions Related to the Pandemic Questionnaire]

14. Recall the last time you looked for or received news about the current COVID-19 situation. How did you feel upon reading or hearing the news?
    - Feeling positive – it's going to be ok
    - Feeling negative – it's not going to be ok

15. Do you consider the current COVID-19 situation a threat to your health or safety?
    - Yes
    - No

16. Do you think the news you receive correctly convey what's happening about the COVID-19 situation?
    - Yes
    - No

[Actions Related to the Pandemic Questionnaire]

17. Are you actively taking actions about the current COVID-19 situation?
    - Yes
    - No

18. What actions have you taken about the current COVID-19 situation? (check all that apply)
    - Social distancing
    - Working from home
    - Distance learning from home
    - Staying at home, unless to get food and seek medical assistance and other essential activities
    - Other, please specify ______
    - Other, please specify ______

19. You might use some the following applications and systems to virtually stay connected with friends, family members, and your social network. If so, please estimate how much time you spend on these systems nowadays on a daily basis. 0 hours would mean that you don't use that application.
    - Social media, including Facebook, Twitter, WhatsApp, etc. _____ hours
    - Telecommunication, including calling, texting, FaceTime, Skype, etc. _____ hours
    - Email _____ hours
    - Other, please specify _____ hours
    - Other, please specify _____ hours

20. How important it is for you to stay connected with friends, family members, and your social network?
    - Extremely important
    - Very important
    - Moderately important

- Slightly important
- Not at all important

[Demographic Questionnaire]

21. What is your gender?
    - Female
    - Male

22. What is your age?
    - ______ years old

23. Your primary residence is in
    - Urban area
    - Suburban area
    - Rural area
    - Other, please specify _______

24. In which State do you live now?
    - ______

25. What is your race and ethnicity (check all that apply)?
    - American Indian/Alaska Native
    - Asian
    - Black
    - Hispanic/Latino/Spanish origin
    - Native Hawaiian/Other Pacific Islander
    - White
    - Some other race or origin, please specify ______

26. What is the highest degree or level of education you have completed?
    - Less than 9th grade
    - Some high school
    - High school graduate
    - Some college
    - College (associate or Bachelor's) degree
    - Graduate degree

27. What is your annual household income?
    - <$25,000
    - $25,000-$45,000
    - $45,000-$70,000
    - $70,000-$110,000
    - >$110,000

28. What is your current employment status? (check all that apply)
    - Full-time
    - Part-time/casual

- Self-employed
- Student
- Unemployed
- Other, please specify ______

[Attention Check Questions]

a. Please indicate the current year: __________

b. If you are reading this, please selection option 3
   - Option 1
   - Option 2
   - Option 3
   - Option 4

c. Please indicate the current month: ___________
